# Hepatocellular-Carcinoma-Derived Organoids: Innovation in Cancer Research

**DOI:** 10.3390/cells13201726

**Published:** 2024-10-18

**Authors:** Carlo Airola, Maria Pallozzi, Eleonora Cesari, Lucia Cerrito, Leonardo Stella, Claudio Sette, Felice Giuliante, Antonio Gasbarrini, Francesca Romana Ponziani

**Affiliations:** 1 Liver Unit, Centro Malattie dell’Apparato Digerente (CEMAD), Medicina Interna e Gastroenterologia, Fondazione Policlinico Universitario Gemelli IRCCS, 00168 Rome, Italy; airollac@gmail.com (C.A.); mariapallozziucsc@gmail.com (M.P.); lucia.cerrito@policlinicogemelli.it (L.C.); leonardo.stella@guest.policlinicogemelli.it (L.S.); antonio.gasbarrini@unicatt.it (A.G.); 2 GSTeP Organoids Research Core Facility, Fondazione Policlinico A. Gemelli, 00168 Rome, Italy; eleonora.cesari@unicatt.it (E.C.); caudio.sette@unicatt.it (C.S.); 3 Department of Neuroscience, Section of Human Anatomy, Catholic University of the Sacred Heart, 00168 Rome, Italy; 4 Department of Surgery, Fondazione Policlinico Universitario A. Gemelli IRCCS, 00168 Rome, Italy; felice.giuliante@unicatt.it; 5 Dipartimento di Medicina e Chirurgia Traslazionale, Università Cattolica del Sacro Cuore, 00168 Rome, Italy

**Keywords:** drug sensitivity, hepatocellular carcinoma, liver organoids, tumor microenvironment

## Abstract

Hepatocellular carcinomas (HCCs) are highly heterogeneous malignancies. They are characterized by a peculiar tumor microenvironment and dense vascularization. The importance of signaling between immune cells, endothelial cells, and tumor cells leads to the difficult recapitulation of a reliable in vitro HCC model using the conventional two-dimensional cell cultures. The advent of three-dimensional organoid tumor technology has revolutionized our understanding of the pathogenesis and progression of several malignancies by faithfully replicating the original cancer genomic, epigenomic, and microenvironmental landscape. Organoids more closely mimic the in vivo environment and cell interactions, replicating factors such as the spatial organization of cell surface receptors and gene expression, and will probably become an important tool in the choice of therapies and the evaluation of tumor response to treatments. This review aimed to describe the ongoing and potential applications of organoids as an in vitro model for the study of HCC development, its interaction with the host’s immunity, the analysis of drug sensitivity tests, and the current limits in this field.

## 1. Introduction

Hepatocellular carcinomas (HCCs) are characterized by peculiar tumorigenesis, a specific tumor microenvironment (TME), a highly expressed vascularization, and a marked resistance to chemotherapies. Overall, these features make it difficult to study a tumor as heterogeneous as HCC; due to the lack of information on microscopic and molecular features, the diagnosis relies mainly on radiologic findings. For decades, the study of HCC biology has been based on two-dimensional (2D) cell culture systems and transgenic mouse models. While they have helped advance the knowledge of the disease, both models also present limitations, particularly concerning the loss of information on cell-to-cell signaling, impairing the physiological cell behavior (i.e., proliferation, migration, and apoptosis), and the reproduction of the complex mutational status of individual HCCs [1,2]. Additionally, the monolayer growth and absence of extracellular matrix components hinder cell-to-cell and cell-to-matrix interactions, which are vital aspects of tumor biology [3,4,5].

For the study of such a complex and highly heterogeneous malignancy, establishing a new method that can recapitulate the genomic features and drug sensitivity of the patient is extremely important.

Three-dimensional (3D) cultures more closely mimic the in vivo environment, influencing factors such as the spatial organization of cell surface receptors and gene expression [6]. Furthermore, compared to other experimental models, such as animal models, organoids clearly maintain human biological and histological traits. For instance, despite a high degree of similarity, there are several cellular and molecular differences between mice and humans. In cancer research, this limitation has been partially addressed by the use of patient-derived tumor xenografts. Nonetheless, the advent of organoid tumor models has revolutionized our understanding of tumor pathogenesis and progression by faithfully replicating the original genomic, epigenomic, and microenvironmental landscape of cancers, with the possibility of discovery of early diagnostic markers and prognostic factors [7].

This review aims to describe the ongoing and potential applications of liver organoids in the clinical management of HCCs, focusing on tumor characteristics and aggressiveness and their correlation with the patient’s prognosis and the response to systemic therapies.

## 2. HCC Organoids: Methods of Production and Differences from Healthy Liver Organoids

The rapid advances in the organoid field over the last 10 years are the result of decades of work aimed at better understanding adult stem cell (ASC) function, the self-organization of dissociated tissues, and extracellular matrix (ECM) biology [8]. In 2017, building upon the initial protocols for expanding adult liver ductal progenitor cells, Broutier et al. achieved a breakthrough by generating the first patient-derived HCC organoid model using tissue specimens from eight patients undergoing liver resections [9]. The problem of short-term expansion of ASC-derived organoids was overcome by setting culture conditions able to reproduce an ECM environment with a combination of growth factors that are essential during liver development and regeneration, such as hepatocyte growth factor (HGF), epithelial growth factor (EGF), fibroblast growth factor (FGF), and R-Spondin1 [10,11]. This allowed for the long-term expansion of adult healthy liver organoids [12]. The following year, the same group published the first protocol to generate organoids from primary liver cancer biopsies [9], which was then adapted by Nuciforo et al., allowing for the use of tissues derived from patients who present with more advanced disease stages, as opposed to candidates for surgical resection [13]. In 2022, Narayan et al. also created organoids from both liver tumor (primary liver tumors or metastasis) and adjacent nontumor tissue [14]. The procedure was comparable, as described by Broutier et al.; in particular, tumor organoids were successfully cultured in both hepatocytes and cholangiocytes media as nontumor ones. However, there are still few but significant differences between the protocols to obtain healthy liver organoids or tumoroids. Unlike healthy tissue, which can be stored at 4 °C for up to 48 h before processing, cancer tissues must be processed within 20 min after collection to have a better success rate, which today is approximately 30% for liver tumoroids [9,13] and 80% for healthy liver organoids. Due to the greater stiffness of HCC tissues, a difference in the protocol used for healthy tissue was the longer enzymatic digestion time for tumor biopsy, which also reduced the number of viable healthy contaminating cells. Unlike healthy tissue digestion, which requires a maximum of 90 min, the tumor tissue needed an incubation time from 2–5 h to overnight in the digestion solution, composed of a blend of collagenase D or IV and DNAse I, depending on the degree of liver fibrosis and the dimension of the biopsy. For very small samples, such as needle biopsy, the estimated time of digestion was 2–4 min [13]. The digestion stopped when the suspension contained 80–100% single cells for the healthy tissue. For the tumor tissue, the digestion stopped when no pieces of tissue were left, but it is important to avoid complete digestion to single cells, because the preservation of cell–cell contact enhances derivation efficacy [15]. After digestion, healthy or tumor cells were seeded in basement membrane extract (BME2). The formation of healthy liver organoids or tumoroids was possible due to the use of an optimized culture medium. On top of the backbone for media components, there is the use of advanced Dulbecco’s modified Eagle’s medium (ADMEM)/F12 that is added with plenty of the small molecules and biologicals required to mimic the stem cell niche signaling:

- Growth factors: EGF, HGF, FGF-10, and gastrin, signaling factors important for liver development and to prolong the survival time of liver organoids;
- B27 and N2 supplements that suppress cell differentiation;
- Amino acids: l-glutamine, which participates in the cellular energy metabolism and intercellular adhesion [16]; N-acetyl-l-cysteine, which is an effective antioxidant and free radical scavenger and regulates cell proliferation, differentiation, and apoptosis [17]; and nicotinamide, which is important for the self-renewal of HCC stem cells [10];
- Inhibitors: A83-01, an inhibitor of mesenchymal cells; Noggin, an inhibitor of bone morphogenetic protein (BMP) 4 and BMP-7; and Y27632, an inhibitor of ROCK, which together support stem cell survival and proliferation;
- Conditioned media with R-Spondin1 and Wnt3a is essential for liver homeostasis and regeneration but is not used in the tumoroid culture medium;
- Forskolin, an adenylate cyclase activator, which supports liver organoids expansion;
- Thanks to these adjustments to the published protocols for the generation of healthy organoids, it is now possible to gain insights into tumor progression and apply personalized medicine through the use of liver cancer organoids.

## 3. HCC Tumorigenesis Reproduced in Organoids

Liver tumor organoids have emerged as invaluable tools for deconstructing the complex mechanisms underlying HCC initiation. These three-dimensional in vitro models recapitulate the tumor microenvironment and can be genetically manipulated using techniques such as retroviral/lentiviral transfection or clustered regularly interspaced short palindromic repeats/CRISPR-associated protein 9 (CRISPR/Cas9), allowing for the elucidation of the functional roles of specific oncogenes [18]. Lam et al. [19] employed this approach to investigate the expression of the well-characterized tumor suppressor protein 53 (p53) and its gene TP53. Their study utilized three distinct liver organoids: one completely knockout for TP53, another harboring a missense mutation (TP53 R249S), and a wild-type control. Compared to the well-defined ductal structures observed in wild-type organoids, TP53 ablation resulted in the formation of compact clusters. Interestingly, the R249S mutation led to an atypical growth characterized by bud-shaped protrusions, strikingly resembling the morphological features of HCC organoids. Histological analysis further revealed a shift from single-layered structures in wild-type controls to multilayered or dysplastic formations in TP53-manipulated organoids. Notably, these morphological alterations were associated with a significant increase in cluster differentiation (CD) 44 and CD-133 expression, the established markers of cancer stem cells (CSCs) [19]. De Crignis et al. [20] further utilized organoids to identify the genes associated with HCC development in cirrhotic patients with chronic hepatitis B virus (HBV) infection. Notably, organoids derived from infected patients who had HBV DNA integrated in their genome, while lacking active viral replication, and displayed an enrichment of genes involved in metabolic pathways compared to healthy donors. Interestingly, cyclin A1 (CCNA1) and stathmin 2 (STMN2), both upregulated in these organoids derived from nontumor-infected patients, have been previously linked to HCC development [21,22]. Furthermore, when compared to the gene expression data available from The Cancer Genome Atlas Liver Hepatocellular Carcinoma (TCGA-LIHC) database, the gene expression profile of organoids derived from infected patients resembled an early cancer signature [20]. In addition, a marked downregulation of tumor suppressor genes, such as WNK lysine deficient protein kinase 2 (WNK2), RUN and SH3 domain containing 2 (RUSC2), cytochrome P450 3A4 (CYP3A4), and regucalcin (RGN), was reported in organoids derived from infected patients compared to healthy controls [20]. These findings collectively underscore the utility of organoids as reliable ex vivo models to investigate HCC initiation specifically in the context of chronic HBV infection-related cirrhosis [20]. Another intriguing perspective is the use of organoids for early HCC diagnosis. By studying the initial stages of tumorigenesis using organoids, it could be possible to identify early biomarkers that could significantly improve diagnostic rates [23]. Another potential application is the identification of genes associated with HCC progression and the development of aggressive behavior. Studies have shown a correlation between higher brain-expressed X-linked gene 2 (BEX2) expression in HCCs and poor prognosis [24]. Analysis of organoid formation using the Huh-7 HCC cell line transfected with a BEX2-expressing vector revealed a significant increase in the total organoid area compared to controls transfected with an empty vector [24]. This suggests a potential role for BEX2 in promoting organoid growth. Nonetheless, BEX2 seems to play a crucial role in maintaining the dormant CSCs in HCCs [24]. Similarly, the role of kinesin family member 15 (KIF15) has been explored using patient-derived organoids. KIF15 downregulation significantly reduces organoid growth, and its overexpression has been linked to a more aggressive tumor phenotype, including microvascular invasion, incomplete tumor capsulation, and larger tumors [25], suggesting a complex role in HCC tumorigenesis, involving the promotion of CSCs through a phosphoglycerate dehydrogenase (PHGDH)-mediated reactive oxygen species (ROS) alteration pathway [25].

These findings underscore the versatility of organoids in elucidating the interplay between specific genes and tumor progression, highlighting the role of CSCs, which better grow up in a three-dimensional culture [26].

## 4. Liver Cancer Stem Cells in HCC Organoids

CSCs have been implicated in tumorigenesis since their discovery in the 1970s. These elusive cells, with self-renewal and differentiation capabilities similar to normal stem cells, contribute to tumor heterogeneity and aggressiveness [27]. Although their phenotypic characterization through molecules such as the Epithelial cell adhesion molecule (EpCAM), leucine-rich repeat-containing G-protein-coupled receptor 5 (Lgr5), CD-133, and CD-24 is well-defined, little is known about CSCs’ origin [28]. The transformation of liver stem/progenitor or the dedifferentiation of mature hepatocytes or biliary cells can be potential sources [28]. Organoids have emerged as powerful tools to clarify the biology of CSCs in HCCs. Studies using HCC Huh7 cell-line-derived organoids shed light on the interplay between epithelial–mesenchymal transition (EMT) and stemness maintenance [29]. These models revealed that the upregulation of the isoform δ of phosphoinositide-3-Kinasi (PI3K) promotes HCC cell plasticity, leading to the coexpression of epithelial and mesenchymal markers alongside pluripotency genes, such as sex-determining region Y-box 2 (SOX2), octamer transcription factor 4 (OCT4), and Nanog homeobox (NANOG) [29]. Furthermore, organoids recapitulated the intrinsic lumen-forming capacity of stem cells [30]. Interestingly, PI3K inhibition triggered dedifferentiation, characterized by the loss of epithelial markers with the rise in mesenchymal genes, and the formation of polarity-compromised organoids [29]. Both these processes, one leading to the coexpression of epithelial and mesenchymal markers alongside pluripotency genes, and the other leading to the loss of epithelial ones, are influenced by the TGFβ signaling pathway and contribute to tumor development by promoting invasiveness, motility, metastasis, and drug resistance [31]. Another molecule usually overexpressed in the aggressive form of HCCs is the CRIPTO protein [32,33]. Notably, CRIPTO+ HCC cell-line-derived organoids exhibit a marked increase in EMT compared to CRIPTO-downregulated counterparts [32]. Nonetheless, CRIPTO overexpression in HCC tissue has been associated with shorter overall survival and time to recurrence in 205 patients with HCC treated with surgical resection [34].

## 5. HCC Organoids: Clinical Applications

**Figure 1:**
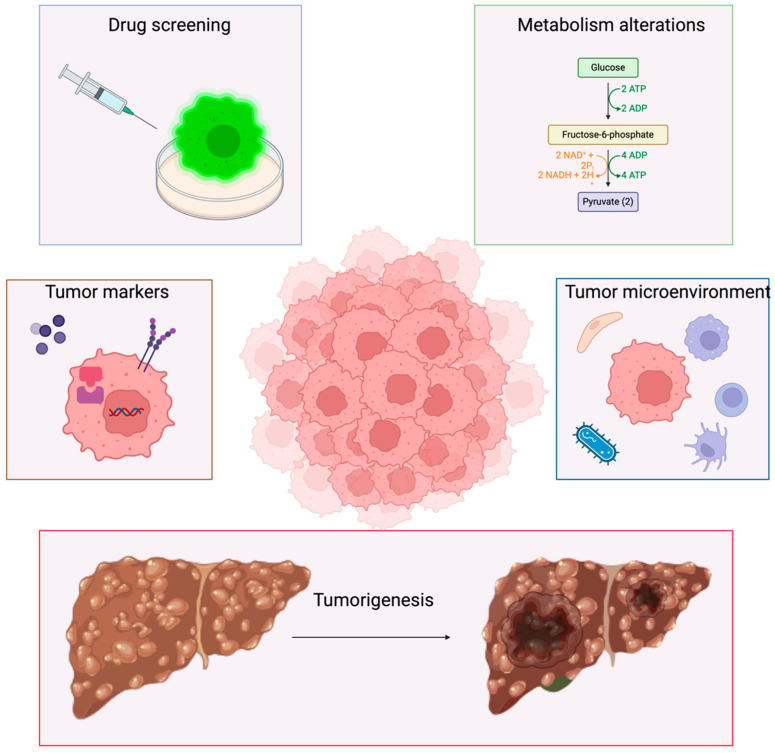
Clinical applications of HCC-derived organoids. Hepatocellular carcinoma liver organoids’ current applications: Considering the complexity of these three-dimensional models, organoids are increasingly used in cancer research in order to mimic real-life cell-to-cell interactions in tumors. Concerning hepatocellular carcinoma, organoids are useful for the in vitro and in vivo study of cancer cell and cancer stem cell behavior, to evaluate cell signaling, and to unravel the specific alterations in inflammatory, metabolic, and proliferative pathways that lead to tumorigenesis, growth maintenance, immune suppression, angiogenesis, and the mechanisms of resistance and tumor escape through the replication and analysis of the tumor microenvironment. Organoids can also be used for drug screening, for investigating the key drivers of HCC development, and to identify markers of aggressiveness.

The main clinical applications of HCC organoids in oncology concern the identification of markers of aggressiveness, the evaluation of underlying metabolic pathways, TME, and drug screening tests (Figure 1).

### 5.1. Markers of HCC Aggressiveness

The close link between the aggressive behavior of HCC organoids and the original tumors highlights the potential of identifying specific molecules associated with tumor aggressiveness. For instance, the neuronal cell adhesion molecule (NRCAM) is a molecule involved in the activation of mitogen-activated protein kinases (MAPK) and PI3K/protein kinase B (Akt) pathways, both implicated in tumorigenesis [35,36]. Analysis of HCC organoids revealed high NRCAM expression in HCC tissue compared to healthy controls, with higher levels correlating with poorer prognosis [37]. Serum analysis showed increased NRCAM levels in HCC patients, comparable to alphafetoprotein for diagnosis. Importantly, NRCAM levels were even higher in metastatic HCCs, suggesting moderate predictive accuracy for metastasis [37]. Using mice CSC-derived HCC organoids, researchers found that NRCAM activation was associated with metastatic potential and the upregulation of MYC, a hallmark of cancer stem cells [37]. Furthermore, NRCAM knockdown significantly reduced the invasion and migration rates in these models. Transcriptomic analysis confirmed that NRCAM is highly expressed in CSCs and decreases with differentiation into mature HCC cells. NRCAM expression changes correlated with alterations in WNT/beta catenin (β-catenin) pathway signaling and Matrix metalloprotease (MMP) 3/7/14 expression [37]. Conversely, protein methyltransferase 6 (PRMT6) is frequently downregulated in HCCs, and its expression correlates with less aggressive features [38]. PRMT6 knockout mice displayed a more aggressive course of chemically induced HCCs. Patient-derived HCC organoids were used to analyze the effects of PRMT6 silencing, revealing a marked increase in tumor initiation, metastasis, and resistance to therapy. Transcriptome and protein–protein interaction studies also revealed an overexpression of genes involved in rat sarcoma (RAS) signaling. Furthermore, in these models, PRMT6 interacts with Raf-1 proto-oncogene, serine/threonine kinase (RAF1), inhibiting its interaction with RAS and reducing the downstream mitogen-activated protein kinase kinase (MAPK) signaling [38]. A subsequent study using patient-derived HCC organoids showed that decreased PRMT6 activity correlates with a metabolic shift towards increased anaerobic glycolysis (Warburg effect) in cancer cells [39]. However, PRMT6 silencing also activates MAPK signaling and enhances the stemness properties of CD-133+ liver CSCs [38]. In another patient-derived HCC organoids model, it has been found that peroxisome proliferator-activated receptor gamma coactivator 1-alpha gene (PPARGC1A) overexpression induced by lentivirus reduced the growth of organoids by inhibiting Wnt/β-catenin signaling and BAMBI production [40]. It is noteworthy that higher BMP and activin membrane bound inhibitor (BAMBI) messenger RNA expression in HCCs has been associated with a poor prognosis [41].

### 5.2. Metabolic Alterations Linked with HCCs Revealed by Organoids

Mitochondrial dysfunction, characterized by defects in oxidative phosphorylation and increased reactive oxygen species production, is implicated in HCC development [42]. Indeed, studies have shown that alterations in mitochondrial ribosomal protein L12 (MRPL12), essential for mitochondrial ribosome biogenesis, significantly reduce tumor cell growth in HCC cell lines and patient-derived organoids [42,43]. Another metabolic feature associated with cancer stem cells is lipid desaturation [44]. HCC organoids derived from EpCAM+ cells displayed high MYCN expression and tumor growth. However, lipid desaturation inhibition downregulated MYCN and reduced organoid proliferation; however, introducing unsaturated fatty acids restored these effects [45]. Interestingly, increased fatty acid desaturase 1–3 activity is observed in nonalcoholic fatty liver disease (NAFLD)-related HCCs [46]. Organoids can also be integrated with other preclinical models to gain a more comprehensive understanding of HCC metabolism. For example, Zhang et al. investigated urea cycle alterations in HCCs. They found that the reduced expression of solute carrier family 25 member 15 (SLC25A15), a gene involved in the urea cycle, correlated with advanced stage and poor prognosis in HCC patients [47]. In vitro studies using 2D cell cultures confirmed that SLC25A15 regulates HCC cell proliferation and lipogenesis through glutamine metabolism [47]. Furthermore, organoids derived from patients with high SLC25A15 expression displayed greater sensitivity to glutamine deprivation than those with low expression [47]. In vivo studies using mice models demonstrated that low SLC25A15 tumors were less responsive to immune checkpoint inhibitor (ICI) therapy, potentially due to reduced programmed death ligand 1 (PD-L1) expression mediated by glutamine metabolism [47].

### 5.3. Tumor Microenvironment and HCC Organoids

HCC development is heavily influenced by the tumor microenvironment, a complex ecosystem composed of various cell populations, such as immune cells, hepatic stellate cells, endothelial cells, and fibroblasts [48]. The ECM and the intricate interactions within the TME are critical for HCC initiation, progression, metastasis, and response to treatment [48,49]. Furthermore, tumor heterogeneity is highly dependent on the specific composition of the TME [49]. Organoids offer a valuable model for studying the interplay between HCC cells and all the other components of the TME [50]. For instance, researchers have used patient-derived HCC organoids to analyze patients’ immune responses against their own cancer. These organoids were cocultured with autologous dendritic cells (DCs) that were activated to stimulate T cells. Organoids derived from patients with a more elevated expression of high-affinity neoantigens (new tumor-specific antigens) on the major histocompatibility complex I (MHC I) displayed a significant increase in the antitumor activity of CD-39+ CD-8+ tumor-infiltrating lymphocytes (TILs). This finding correlated with a higher overall survival rate in these patients [51]. To understand the role of different cell types in HCC development, Qui et al. created multicellular HCC organoids incorporating human HCC cell lines (Huh7, HepG2) and human-induced pluripotent stem cells (iPSCs) [52]. These organoids were then implanted with high engraftment rates in mouse models. By controlling the number of cell populations, the study revealed that adding iPSC-derived mesenchymal cells or endothelial cells to Huh7-Luci cells promoted tumor growth [52]. Although a comprehensive investigation using HCC organoids to map the intricate interplay between the ECM and tumors is still lacking, some studies have shed light on this complex interaction. Van Tienderen et al. employed a decellularization process on HCC surgical samples and adjacent nontumor tissue to isolate HCC-associated ECM. Subsequent proteomic analysis revealed that several proteins, including extracellular matrix protein 2 (ECM2), MATN3, KIT ligand (KITLG), and proplatelet basic protein (PPBP), were overexpressed in the tumor-derived ECM [53]. Interestingly, ECM2 overexpression has been linked to a poorer prognosis in HCC patients [54]; whereas, MATN3 upregulation has been associated with a reduced response to chemotherapy [55]. The decellularized ECM was also used to create a hydrogel for culturing tumor organoids [53]. This innovative model holds promise for further investigations on the role of the ECM in HCC tumorigenesis. Another study compared cell–cell contact in cocultures of endothelial cells from patient-derived HCC organoids and spheroids from Huh7 cell lines, showing that organoids have a superior ability to establish cell–cell contacts. This HCC–endothelial cell interaction led to the upregulation of monocyte chemoattractant protein-1 (MCP-1) and chemokine C-X-C motif ligand (CXCL) 8 and 16, all recognized as angiocrine signaling molecules involved in immune cell recruitment, cancer stem cell maintenance, and tumor aggressiveness [56]. Furthermore, when macrophages were introduced to the HCC–endothelial coculture, they polarized towards an inflammatory and proangiogenic phenotype [56].

### 5.4. Tumor Organoids and Drug Screening

HCCs are characterized by a high resistance to chemotherapies, and for over a decade, only tyrosine kinase inhibitors (TKIs) resulted in superior-to-best supportive care in the management of unresectable, advanced HCC cases [57,58]. From 2020, the combination of immunotherapy and antiangiogenic agents has improved patients’ overall survival compared to TKIs, reshaping the treatment scenario [59]. Despite these advances in the treatment approach, HCCs remain a highly drug-resistant tumor, so models to evaluate the drug sensitivity of HCCs are a hot topic in medicine. Organoids have been used to test several anticancer drugs, bringing information on sensitivity, as well as mechanisms of resistance. Broutier et al. obtained organoids from eight different patient-derived primary liver-cancer-derived organoids, including poorly differentiated to well-differentiated HCCs, which were tested against 29 anticancer drugs. Among these, taselisib, gemcitabine, AZD8931, SCH772984, and dasatinib could inhibit tumor growth in all the models. Also, specific gene mutations in HCC organoid models were associated with drug sensitivity or resistance: LGK974, which was one of the screened drugs, inhibited HCC organoids that presented Wnt pathway mutations, while it was ineffective in models carrying the catenin beta 1 (CTNNB1) mutation. Moreover, many models demonstrated sensitivity to gemcitabine and to the inhibition of MAPK-1 and MAPK-3 pathways, and similar results were observed when translating the experiment to an animal model [9]. Also, the role of the TME must be evaluated in drug screening, since it may lead to resistance against therapy. Indeed, in the study of Liu et al., mouse-liver-derived organoids containing cancer-associated fibroblasts (CAFs) exposed to sorafenib, regorafenib, and 5 fluorouracil showed a poor response compared to samples without CAFs. Similar results were obtained in patient-derived HCC organoid models exposed to a medium enriched with CAFs [60].

#### 5.4.1. Sorafenib

Li et al. developed 27 patient-derived organoids to test up to 129 Food and Drug Association (FDA)-approved anticancer drugs, observing that they were not equally effective for each organoid; only 7 of them demonstrated moderate antitumor activity, with only 2 among these (panobinostat and bortezomib) being previously tested in vivo as systemic chemotherapy in liver cancer. As regards sorafenib, it showed efficacy in the majority of the organoids but not in all of them. Since multiple models were established from the same primary tumor, this study demonstrated that organoids derived from the same patient may be sensitive or resistant to a drug depending on the part of the tumor from which the tissue was obtained. This illustrates the difficulty of recapitulating a realistic model of HCCs, as not only interpatient but also intrapatient variability should be taken into account [61]. Sorafenib was also tested in a study by Nuciforo et al., who exposed patient-derived HCC organoids at increasing concentrations of this therapeutic agent, observing a reduction in HCC growth in a dose-dependent manner, with the half-maximal inhibitory concentration values within a 2.5-fold range from 2 to 5 mM and an unexplainable difference in responses among the available organoids. Unfortunately, a direct correlation between in vitro and in vivo response was not observed, since these HCC organoid models were obtained from patients that did not receive sorafenib as treatment [13]. A possible explanation may be associated with differences in gene expression between the models. Cao et al. created organoids from nine allograft tumors and four primary tumors obtained from patients and tested sorafenib and regorafenib in these models. Interestingly, TKIs inhibited tumor growth, but with several differences in responsiveness among the organoids. As observed before, some mutations may cause resistance or responsiveness; a lower expression of Oct4 and a higher expression of Sox-9 were associated with TKIs sensitivity [62]. Another study reported that the activation of the N-methyl-D-aspartate receptor (NMDAR) caused the growth of HCCs with poor response after sorafenib administration. Adding ifenprodil, an inhibitor of NMDAR, to sorafenib, this pathway was blocked, leading to a significant reduction in patient-derived HCC organoid growth in vitro [63]. Similarly, drugs that act on the Hippo/YAP pathway involved in tumor replication, such as vitepofin, were able to restore antitumor response in HCC organoids derived from patients resistant to sorafenib [64]. It is also known that CRIPTO, a protein encoded by the teratocarcinoma-derived growth factor-1 (TDGF-1) gene, is associated with poor response to sorafenib. Using a cell-line HCC organoid model, Karkampouna et al. showed that the association of sorafenib and doxorubicin with a CRIPTO inhibitor improved antitumor response [32]. Another mechanism of resistance to sorafenib was associated with the sonic hedgehog (SHH) pathway; when added to patient-derived HCC organoids, the SHH inhibitor GANT61 and sorafenib improved antitumor response [65]. Targeting the mammalian target or rapamycin (mTOR) pathway was another way to overcome sorafenib resistance in patient-derived HCC organoids [66]. Finally, in another study with sorafenib-resistant patient-liver-derived organoids, the transcriptomic analysis revealed that 37 genes, including minichromosome maintenance complex component 6 (MCM6) and ribosome biogenesis regulator 1 (RRS1), were upregulated, while 207 genes, including TP53INP2 and MYH14, were downregulated, and genes associated with proliferation and epithelial-to-mesenchymal transition were overexpressed [66].

#### 5.4.2. Lenvatinib

Low frizzled class receptor 10 (FZD10) expression is associated with longer survival after lenvatinib treatment, while higher levels are likely to indicate treatment failure. Patient-derived HCC organoids expressing high FZD10 levels showed resistance to lenvatinib, while lower levels were associated with increased survival. FZD10 is a crucial activator of the MAPK signaling pathway, which is associated with lenvatinib resistance and relapse following lenvatinib administration. Knockdown of c-Jun or β-catenin levels leads to FZD10 reduction, increasing antitumor response. The FZD10 inhibitor resensitized the organoids to lenvatinib, lowering c-Jun, p-MAPK-1/3, and Ki67 expression. Adding the FZD10 inhibitor and lenvatinib in vivo favored growth inhibition [67]. In another model, a histone deacetylases (HDAC) inhibitor overcame the resistance to lenvatinib, upregulating phosphatase and tensin homolog deleted on chromosome 10 (PTEN) and inhibiting AKT signaling. In vitro, the association of lenvatinib plus one of those drugs acts synergistically to inhibit patient-derived HCC organoid growth [68].

#### 5.4.3. Immunotherapy

Studies testing immunomodulating agents in HCC organoids are scant. The only data published involved patient-derived organoids cocultured with or without mesenchymal stem cells (MSCs), peripheral blood mononuclear cells (PBMCs), or CAFs from HCC patients receiving an anti-PD-L1 agent (atezolizumab); the expression of high levels of CD-38 was associated with resistance to treatment, while no differences in terms of response were associated with the expression of PD-L1. Moreover, the presence of MSCs or CAFs enhanced immunotherapy resistance (Table 1) [69].

## 6. Limitations

Although organoids have changed cancer research thanks to their innumerable applications and potentialities, this technology still faces numerous limitations that prevent its widespread application.

### 6.1. Cost and Complexity of Organoid Cultures

Firstly, unsurprisingly, these cultures are expensive due to the expertise and the equipment required for their production. The origin of the tumor samples is not uniform, since they may be obtained from primary tumors, from circulating tumor cells, or retained from liquid effusion. Moreover, samples from different parts of the same tumor can give rise to completely different organoids, as happens in 2D cultures [70]. The nature of the culture may also be established from complete tissue dissociation into single cells that are encapsulated in a matrix support. Otherwise, it may be established by dissociating the tumor sample into small fragments through enzymatic or mechanic methods or inducing critical mutations into a healthy single cell or through the reprogramming of induced pluripotent stem cells [71]. These initial passages lack of a standardized protocol and may lead to different results in studies.

### 6.2. Variability in Culture Medium and Extracellular Matrix (ECM)

The maintenance of cell lines and the replicability of the culture medium are pivotal in order to reduce the intermodel variability. Medium cocktails may suffer from batch-to-batch variability with different activity levels of target proteins; in addition, they contain factors that may influence the cancer organoid phenotype and its drug sensitivity. In particular, in many cancer models, fetal bovine serum (FBS) is added to the medium culture, as it contains several growth factors, including hormones, nutrients, and matrix signaling molecules that help the establishment of the 3D culture. Conversely, it is not standardized, and the variability in concentration of its components, the probability of immunogenic reactions, and the risk of viral or bacterial contamination are major concerns regarding its administration [71,72,73,74]. Similarly, the extracellular matrix, which is pivotal for the spatial organization of cancer organoids, may increase immunogenic risk and alter the reliability of the model, as it is often derived from animals and contains proteins in variable concentrations, leading to batch-to-batch variability. Matrigel, one of the most used extracellular supports, is obtained from Engelbreth–Holm–Swarm mouse sarcoma cells and contains many proteins able to modify cancer cell phenotype. Moreover, these supports often lack tunability to resemble the properties of the real tumor extracellular matrix; in particular, tumor organoids obtained from adult stem cells utilize a simplified extracellular matrix that does not express structural stiffness and does not mimic the compartmentalization of the real ECM often reported in HCCs, which is fundamental to the interaction between tumor cells and stromal cells [75,76,77]. Contaminant healthy liver cells are observed in some tumor models, so a continuative assessment of purity of the organoid must be performed: to remove these cells, it is possible to modify the composition of the culture medium, removing factors that are essential for the growth of normal cells [78,79,80].

### 6.3. HCC Organoids Establishment Compared to Other Tumor Organoids

The mean success rate of cancer organoid generation is reported to be >70%, with a peak of 90% in some colorectal cancer models [81,82,83,84]. Conversely, regarding HCC organoids, in the study of Nuciforo et al., the success rate decreased to 26% of cases. Also, they observed that samples derived from cancer cells with low-grade histological differentiation led to the successful generation of HCC organoids more frequently, probably due to the higher replicative activity associated with immature cells [13]. Similarly, Broutier et al. reported a strict correlation between the original tumor proliferation index and the success rate in organoid production: a replication rate above 5% was associated with a success rate of 100%. These data suggest that histological and replicative features of primary HCCs influence the realization of HCC organoids [9,13]. In order to establish a tumor organoid that perfectly mimics patient-specific cancer heterogeneity, the original samples should cover the spatial–temporal diversity of the tumor. In real life, cancer organoids are obtained from single liver biopsies or from resected tissue, losing information on the intratumor differences or its in vivo temporal evolution. Moreover, some characteristics of the original tumor may influence organoid establishment, such as cancer subtype, the histopathological grade, and previous treatments, causing poor reliability between the primary tumor and the corresponding in vitro model. Creation of multiple organoids derived from different sites of the primary tumor or the development of a large biobank with genomic, metabolomic tumor information is desirable to overcome this limitation [85,86].

### 6.4. Genomic Instability of Tumor Organoids

Another problem is related to the acquisition of de novo mutations in organoid tumor models, despite the long-term expansion in culture, which has been confirmed to be stable after a year of replication [9]. Mutations may affect their reliability with the original in vivo model of cancer, with the loss of patient and tumor genetic signatures. This often happens in tumor organoids with inherent genomic alterations, such as colorectal cancer organoids in the presence of microsatellite instability [87]. One of the more frequently reported mutation occurs in the TP53 gene, enhancing the replication and the aggressiveness of the model [19]. The causes of this phenomenon have not been elucidated yet, but it is suggested that cell–matrix interaction could induce these changes, stimulating tumor cells to acquire antiapoptotic mutations to survive. Even in the absence of genetic mutations, phenotypic instability could be reported in tumor organoids, as it may derive from changes in the culture conditions and from the use of growth factors.

### 6.5. Tumor Organoids Microenvironment

Despite the 3D structure, HCC organoids lack a tumor microenvironment comparable to the primary liver tumor. Lately, combinatory models including nonparenchymal cells belonging to TME are increasing in order to better recapitulate the characteristics of a realistic liver tumor [88,89,90,91]. Many immune cells are usually obtained from the peripheral circulation, such as PBMCs, and they are added to the tumor organoid and exposed to growth factors in culture medium. It is not clear if these PBMCs are comparable to the immune cells located in the liver niche, or if they may cause a bias. Recently, using an air–liquid interface in cancer models derived from colorectal cancer and melanoma, tumor organoids derived from neoplastic epithelium were cocultured with endogenous tumor-infiltrating lymphocytes, derived from the TME. Interestingly, these models were able to express and to maintain the original immune markers of the primary tumor, including the T receptor cell spectrum and response to immune checkpoint inhibitors, resulting in T cell cytotoxicity activation. These promising results may improve the reproducibility of TME in vitro in HCC organoids, but currently, data are scarce [91]. Another crucial point is related to the absence of a strong vascularization in HCC models. Organoids derived from epithelial tumor cells do not express vessels, but angiogenesis is a hallmark of cancer, and it is pivotal to reproduce the invasiveness and the characteristics of the baseline tumor [92]. A hybrid solution is to engraft the tumor organoid into a highly vascularized animal tissue with the aim of stimulating the host vasculature to infiltrate the organoid, providing nourishment, as reported for brain tumor organoids [93,94]. Tumor organoids derived from patient-derived mesenchymal cells and exposed to specific growth factors demonstrated the express markers of angiogenesis [95]. Another approach is obtained by combining cultures of different cells in the same model: HCC organoids cocultured with endothelial cells and fibroblasts expressed markers of vascularization, such as vimentin, vascular endothelial growth factor receptors (VEGFR) 2, hypoxia-inducible factor alpha 1 (HIF-1 alpha), and vascular endothelial growth factor (VEGF). Also, adding CAFs to the culture increases the expression of VEGF. The spatial distribution of VEGF and HIF-1 alpha in the model is essential to guide vascularization and to reproduce the changes observed in in vivo tumorigenesis [96].

### 6.6. The Lack of a Pathological Subset

An additional limitation for the comparability of HCC organoids to reality is the lack of portal hypertension, determined in HCCs by a close relationship between systemic inflammation, endotoxemia, tumor cells, endothelial cells, and their interaction with ECM. Based on all these limitations, nowadays, there is no consensus on how a liver cancer organoid must be cultured, so the variability among the models may generate results and data that are not completely comparable. The application of standardized protocols and the optimization of culture conditions will be mandatory to overcome these limits. The role of biomedicine, the application of bioengineering techniques, and the development of standardized protocols for the culture and maintenance of organoids will be crucial to solve this task [97].

## 7. Conclusions

Organoids hold great potential to advance HCC research, allowing for the creation of complex models that better replicate the intricate network underlying tumor initiation and progression. Beyond their established role in studying CSC origin and behavior, organoids offer a valuable tool for exploring the role of often-overlooked players taking part in the TME, such as immune cells, endothelial cells, and the ECM. This approach could lead to the identification of new diagnostic and prognostic biomarkers, bridging the gap separating HCCs from other tumor types for which much more molecular and genomic data are available. Furthermore, the versatility of organoid models allows for diverse interventions, potentially paving the way for new therapeutic strategies guided by organoid-derived data. Interestingly, organoid models can be a useful tool to accelerate the pre-clinical phase of the selection of anticancer agents in HCCs, revolutionizing the process of development of novel therapies. Organoids also may allow for studying a specific tumor and a patient’s genetic mutations, the molecular expression of transcripts, and the characteristics of the TME; this may help in choosing the drug with the highest probability of response in the single case, which is a prerequisite for the development of a personalized approach to therapy. Ideally, this process could be adapted and modified several times during the course of the disease, adapting therapeutic interventions to the biological plasticity of the tumor as never before. However, these promising and positive aspects are tempered by the fact that the use of HCC organoids is still in the early stage of its scientific development and is affected by many limitations, ranging from the need for laboratories with expertise to the timing of the analysis, that currently make it difficult to apply this approach in real life. Furthermore, large clinical studies are needed to confirm the reliability of the findings and the correspondence with in vivo data. Indeed, despite the inclusion of cells belonging to the TME and the application of specific growth factors, organoids still do not fully recapitulate the TME. To overcome this limit of the model, a step forward is to include the stromal and immune cells of the host, but the intricate relationship between host immunity, vascularization, and tumor cells is still far from mirroring reality. In vitro culture conditions may also influence tumor evolution, altering the selection of specific clones. Not to mention that to recapitulate the changing characteristics of a real tumor, the expression of growth factors and TME characteristics should be constantly reshaped and adapted; as occurs in real life, spatial and temporal changes are always observed during tumorigenesis, leading to the need for high-cost technology, which further limits a wide application in the clinical setting. Despite these limitations, the fascinating idea of using a reproduction of a tumor, which is always available for any test and analysis, represents the dream of oncology, and organoids are an unprecedented opportunity to improve the management of a complex disease with a poor prognosis such as HCC.

## Figures and Tables

**Table 1:** Summary of the studies about the drug sensitivity in HCC-derived organoids previously reported.

	Organoid Type	Organoid Source and Type of Cells	Results	Screened Drugs	Drug Response
Broutier et al. [9]	Healthy liver HCC HCC, CCA CCA	Liver resection + enzymatic digestion Healthy liver cells Primary liver cancer cells	Tumor-derived organoids recapitulated and preserved the histological characteristics of the original tumor tissue ↑ C19ORF48, UBE2S, and DTYMK in HCC organoids	Cisplatin Olaparib KU-55933 5-Fluorouracil Doxorubicin Gemcitibine Axitinib PD-173074 Sorafenib AZD8931 Lapatinib CH5424802 EMD-1214063 Trametinib Dabrafenib SCH772984 Deltarasin MK-2206 Taselisib OSI-02 Vorinostat BIRB Nutlin-3° PD-0332991 LGK974 PORCN LY2109761 GSK126 BIBR-1532 Dasatanib	LGK974 inhibits Wnt-pathway-mutated HCC but not CTNNB1-mutated HCC organoids. AZD8931 inhibits K-RAS WT HCC but not K-RAS-mutated organoids SCH772984 inhibits MAPK1/3 phosphorylation in HCC organoids
Liu et al. [60]	Mice-derived organoid coculture with CAFs Human primary liver cancer organoid coculture with human CAFs Mice or human organoids +/− CAF transplanted in NSG immunodeficient mice	Liver resection + enzymatic digestion Cancer stem cells + CAFs	CAFs promote organoid growth and renewal Coculture organoids + CAFs favor survival of the cancer models in mice through paracrine signaling (involving Lrig1, Muc5ac, CD133, TERT, NANOG in CSCs and gremlin-1 in CAFs)	Sorafenib Regorafenib 5-FU	Medium exposed to CAFs causes drug resistance against sorafenib, regorafenib, and 5-FU in both mice- and human-cancer-derived organoids
Li et al. [61].	Human HCC and CCA	Liver resection + fragmentation Primary liver tumor cells	PDOs express the same markers of the original tumor (EPCAM, LGR5, CK19, AFP, and HepPar1) PDOs recapitulate intratumor and interpatient differences	Drug sensitivity applying NCI set VII that includes 129 FDA-approved cancer drug library	In total, 13/129 drugs showed more than 90% killing across all 27 liver cancer PDO lines In total, 9 drugs were effective, but only two of them were used in real life All PDO lines showed similar IC50 doses, with variable intratumor responses Inter- and intratumor heterogeneity in drug sensitivity
Nuciforo et al. [13]	HCC Liver cells	US-guided needle biopsies of tumor and nontumor tissue + enzymatic digestion	Tumor organoids show same morphology and tumor marker expression of the original tumors Tumor organoids preserve the genetic heterogeneity of the originating tumors	Sorafenib	Sorafenib ↓ organoid growth in a dose-dependent manner Sorafenib IC50 varies by 2.5-fold from 2 to 5 mM
Cao et al. [62]	HCC and CCA from primary mice liver tumor	Enzymatic digestion Primary mice liver tumor	Organoids derived from a single cell were able to replicate and self-renew Organoid transplantation in mice was able to induce tumorigenesis Organoids expressed markers of hepatocytes (e.g., AFP, HNF4ɑ) or/and cholangiocytes (e.g., CEA, CK19, C-KIT, and EpCAM)	Sorafenib Regorafenib	According to sorafenib and regorafenib sensitivity, organoids were divided into 3 groups: - Group 1 sensitive to both; - Group 2 only sensitive to sorafenib; - Group 3 not sensitive to either drug.
Xu et al. [63]	HCC organoids 2D cell cultures Human HCC cell lines	Liver resection HepG2, Hep3B, and HEK293T Human HCC cell line Huh7	PDOs showed markers of HCC	Sorafenib Regorafenib MK-801 IFEN INF ERA SAR131675	NMDAR inhibitors (INF) + sorafenib and lenvatinib inhibit tumor organoid replication IFEN is more selective for NMDAR1/NMDAR2B subunit and acts synergistically with sorafenib on 2D and 3D cell cultures This combination induces unfold protein response, ↓ WNT signaling, and results in G1 phase cell-cycle arrest in HCC cells IFEN plus sorafenib inhibits tumor growth in transplanted PDOs in mice ERA and SAR act on FLT4 and FGF3 similarly to sorafenib and regorafenib
Zhao et al. [64]	Tumor liver cells Nontumor liver cells	Liver resection + collagenase digestion	ACAD is ↓ in HCC organoids, and it is related to poor prognosis ↑ ACADL in HCC organoids causes ↓ Hippo/YAP signaling leading to cell death Reactivation of YAP by XMU-MP-1 diminished the inhibitory effect of ACADL on HCC organoid growth	Veterporfin (YAP inhibitor)	Verteporfin suppressed growth of HCC organoids with low ACADL expression
Wang et al. [65]	HCC patient-derived organoids	Liver resection + enzymatic digestion	HCC PDOs strongly maintained the histological features of the corresponding tumors and responded to drug treatment CD-44 and SHH were associated with HCC organoid invasiveness and aggressiveness	GANT61 Sorafenib	CD-44 and SHH cause sorafenib resistance GANT61 (anti-SHH) + sorafenib restores sensitivity to systemic therapy in vitro and in vivo
Xian et al. [66]	Primary liver cancer organoids	ODX and PDX	HCC-derived organoids show higher success rate (29% vs. 23.7%) Tumor dimension, vascular invasion, high AFP, and upregulation of stemness associated with organoid establishment Organoids recapitulate histology of original tumor tissue	Sorafenib Lenvatinib mTOR inhibitor	Organoids are resistant to lenvatinib but in vivo show sensitivity Sorafenib-resistant organoids show EMT, and stemness mTOR inhibitors restore sorafenib-sensitivity in HCC organoids, possibly via inducing phosphorylated S6 kinase
Wang et al. [67]	PDOs and PDXs	HCC organoids from liver resection and enzymatic digestion HCC cell lines Hep3B, Huh7, and SNU398 Patient-derived xenograft in immunodeficient mice	FZD10 activation mediated by METTL3-dependent N6-methyladenosine methylation of its mRNA FZD10 activates β-catenin/YAP1 and c-Jun pathways	Lenvatinib FDZ10 inhibitor β-catenin inhibitor	FZD10/β-catenin/c-Jun/MAPK axis determines the responses of hepatoma cells to lenvatinib treatment FZD10 or β-catenin inhibitors restored lenvatinib response in resistant organoids Prognostic biomarker of resistance to lenvatinib
Yan et al. [68]	Patient liver cancer organoids	Liver resection + mechanical fragmentation + collagenase digestion	Downregulation of PTEN and upregulation of AKT pathway lead to tumor progression and influence drug sensitivity in HCC organoids	SAHA	PTEN and AKT signaling influences lenvatinib sensitivity SAHA upregulates PTEN and inhibits AKT pathway SAHA + lenvatinib inhibits HCC growth
Zou et al. [69]	Patient- derived organoid	Needle biopsy Coculture with MSC, CAFs and PBMC	MSC, CAFs, and PBMC coculture increases success rate of biopsy-derived PDO culture, accelerates PDO growth, and promotes monocyte survival and differentiation into tumor-associated macrophages	Atezolizumab	CD-38 expression associated with immunotherapy resistance PD-L1 expression does not influence resistance

Abbreviations: CAFs, cancer-associated fibroblasts; NSG, NOD/scid/gamma mouse; CD, cluster differentiation; Lrig, leucine-rich repeats and immunoglobulin-like domains; TERT, telomerase reverse transcriptase; NANOG, Nanog homeobox; CSCs, cancer stem cells; 5-FU, 5 fluorouracyl; CCA, cholangiocarcinoma; EpCAM, epithelial cell adhesion molecule; AFP, alphafetoprotein; PDO, patient-derived organoid; IC50, half-maximal inhibitory concentration; US, ultrasound; CEA, carcinoembryonic antigen; CK19, cytokeratin 19; NMDAR, N-methyl-D-aspartate receptor; FGF, fibroblast growth factor; SHH, sonic hedgehog; PDX, patient-derived xenograft; mTOR, mammalian receptor of rapamycin; EMT, epithelial-to-mesenchymal transition; MEK, mitogen-activated protein kinase kinase; PTEN, phosphatase and tensin homolog deleted on chromosome 10; MAPK, mitogen-activated protein kinases; AKT, protein kinase B; β-catenin, beta catenin; MSC, mesenchymal stem cells; PBMC, peripheral blood mononuclear cells; TME, tumor microenvironment; PD-L1, programmed death ligand 1.

## Abbreviations

Hepatocellular carcinoma (HCC), tumor microenvironment (TME), two-dimensional (2D), adult stem cells (ASCs), three-dimensional (3D), extracellular matrix (ECM), hepatocyte growth factor (HGF), epithelial growth factor (EGF), fibroblast growth factor (FGF), basement membrane extract (BME2), advanced Dulbecco’s modified Eagle’s medium (ADMEM)/F12, bone morphogenetic protein (BMP), clustered regularly interspaced short palindromic repeats/CRISPR-associated protein 9 (CRISPR/Cas9), tumor suppressor protein 53 (p53), cluster differentiation (CD), cancer stem cells (CSCs), hepatitis B virus (HBV) infection, The Cancer Genome Atlas Liver Hepatocellular Carcinoma (TCGA-LIHC), cyclin A1 (CCNA1), stathmin 2 (STMN2), RUN and SH3 domain containing 2 (RUSC2), cytochrome P450 3A4 (CYP3A4), WNK lysine-deficient protein kinase 2 (WNK2), regucalcin (RGN), brain-expressed X-linked gene 2 (BEX2), kinesin family member 15 (KIF15), phosphoglycerate dehydrogenase (PHGDH), reactive oxygen species (ROS), epithelial cell adhesion molecule (EpCAM), leucine-rich repeat-containing G-protein-coupled receptor 5 (Lgr5), epithelial–mesenchymal transition (EMT), phosphoinositide-3-kinase (PI3K), sex-determining region Y-box 2 (SOX2), octamer transcription factor 4 (OCT4), Nanog homeobox (NANOG), neuronal cell adhesion molecule (NRCAM), mitogen-activated protein kinases (MAPK), extracellular signal-regulated kinase (Erk), protein kinase B (Akt), beta catenin (β-catenin), matrix metalloprotease (MMP), protein methyltransferase 6 (PRMT6), rat sarcoma (RAS), Raf-1 proto-oncogene, serine/threonine kinase (RAF1), mitogen-activated protein kinase kinase (MEK), peroxisome proliferator-activated receptor gamma coactivator 1-alpha gene (PPARGC1A), BMP and activin membrane-bound inhibitor (BAMBI), mitochondrial ribosomal protein L12 (MRPL12), metabolic-dysfunction-associated fatty liver disease (MAFLD), solute carrier family 25 member 15 (SLC25A15), immune checkpoint inhibitor (ICI), programmed death ligand 1 (PD-L1), dendritic cells (DCs), major histocompatibility complex I (MHC I), tumor-infiltrating lymphocytes (TILs), induced pluripotent stem cells (iPSCs), extracellular matrix protein 2 (ECM2), KIT ligand (KITLG), proplatelet basic protein (PPBP), monocyte chemoattractant protein-1 (MCP-1), interleukin (IL), chemokine C-X-C motif ligand (CXCL), tyrosine kinase inhibitors (TKIs), cancer-associated fibroblasts (CAFs), catenin beta 1 (CTNNB1), Food and Drug Association (FDA), N-methyl-D-aspartate receptor (NMDAR), teratocarcinoma-derived growth factor-1 (TDGF-1), sonic hedgehog (SHH), mammalian target or rapamycin (mTOR), patient-liver-derived organoids (PLOs), minichromosome maintenance complex component 6 (MCM6), ribosome biogenesis regulator 1 (RRS1), histone deacetylases (HDAC), phosphatase and tensin homolog deleted on chromosome 10 (PTEN), mesenchymal stem cells (MSCs), peripheral blood mononuclear cells (PBMCs), fetal bovine serum (FBS), vascular endothelial growth factor receptor (VEGFR) 2, hypoxia-inducible factor alpha 1 (HIF-1 alpha), vascular endothelial growth factor (VEGF).
